# Supplementary material for: The Role of Telocytes and Telocyte-Derived Exosomes in the Development of Thoracic Aortic Aneurysm
Source: Int J Mol Sci. 2022 Apr 25;23(9):4730. doi: 10.3390/ijms23094730 (PMC9099883; doi:10.3390/ijms23094730)
Supplement: Supplementary file 1 [file ijms-23-04730-s001.zip › ijms-1689483-supplementary.pdf]

Supplemental Information

Supplement Table S1. List of antibodies and working dilutions used in this study.

Antibody name	Source	Cat.no°	Working Dilution **WB**	Working Dilution **ICC**	Working Dilution **IF**
Primary Antibodies					
SM-calponin	Abcam	Ab78491	1:400	1:250	1:100
ACTB	SantaCruz	sc47778	1:500	-	-
ckit/CD117	Abcam	ab32363	1:200	1:200	4.72µg/ml
PDGFR-α	SantaCruz	sc398206	1:400	1:100	1:100
PDGFR-β	Abcam	ab69506	1:600	1:300	1:300
KLF-4	Abcam	ab75486	1:50	1:50	10µg/ml
VEGF-A	Abcam	ab1316	1:100	1:100	1:100
αSMC	Abcam	ab5694	1:500	1:500	1:500
Vimentin	SantaCruz	sc5565	1:200	1:200	1µg/ml
CD133 (EPR20980-104)	Abcam	ab216323	1:600	1:1000	1:1000
CD63	Abcam	ab68418	1:750	-	-
Integrin β–1 (CD29)	Abcam	Ab183666	1:400	1:200	-
HSP90	CellSignaling	7874S	1:800	-	-
CD34 (B6)	SantaCruz	sc74499	1:400	1:50	4µg/ml
TSG101	SantaCruz	sc7974	1:500	-	-
Secondary Antibodies					
AF488GAR	molecular probes	A11034	-	1:1000	1:500
AF546GAM	molecular probes	A11030	-	1:1000	1:1000
AF488GAM	molecular probes	A11029	-	1:1000	1:1000
AF546GAR	molecular probes	A11035	-	1:1000	1:1000
AF488DAG	molecular probes	A11055	-	1:500	1:500
HRP-anti-mouse IgG	CellSignaling	7076S	1:2000	-	-
HRP-anti-rabbit IgG	CellSignaling	7074S	1:3000	-	-
Isotype-control					
purified mouse IgG	BDPharmigen	550878	-	4 µg/ml	4 µg/ml
purified rabbit IgG	Abcam	Ab27478	-	1.89 µg/ml	1.89 µg/ml

BD Pharmigen^{TM} (BD Biosciences), San Jose, CA; Santa Cruz Biotechnologies, Inc., TX, USA; Invitrogen Molecular Probes, ThermoFisher Scientific, Massachusetts, USA; Novus Biogicals, LLC, CO, USA; Dianova GmbH, Hamburg, Germany.

Supplement Table S2. Primer sequences for qPCR

Target	Forward Primer	Reverse Primer
miRNA primer sequences		
RNU6	CGCTTCGGCAGCACATATAC	AGGGGCCATGCTAATCTTCT
SNORD44	TGATGATAAGCAAATGCTGACTG	GAGCTAATTAAGACCTTCATGTTCAG
hsa-mir-21-5p	GCAGTAGCTTATCAGACTGATG	GGTCCAGTTTTTTTTTTTTTTTCAAC
hsa-mir-24-3p	GATCCTGGCTCAGTTCAGCAGGAACA GC	TCGAGCTGTTCCTGCTGAACTGAGCCAG
hsa-mir-143-3p	GCAGTGCTGCATCTCTG	GAACATGTCTGCGTATCTC
hsa-mir-145-5p	GTCCAGTTTTCCCAGGA	GAACATGTCTGCGTATCTC
hsa-mir-146a	GAGAACTGAATTCCATGG	GAACATGTCTGCGTATCTC
hsa-mir-221-3p	GCCGAGAGCTACATTGTCTG	GTCGTATCCAGTGCAGGG
hsa-mir-221-5p	TCCGCGCCCTTGCCCAGACC	GTGCCTGGTGCTCTCTTACC
hsa-mir-222-5p	GGGCTCAGTAGCCAGTGTA	CAGTGCGTGTCGTGGAGT
mRNA primer sequences		
VIM	AGGCAAAGCAGGAGTCCACTGA	ATCTGGCGTTCCAGGGACTCAT
KLF4	CGACGCGCTGCTCCCATCTT	GGCAGTGTGGGTCATATCCA
KIT	TCATCGAGTGTGATGGGAAA	GGTGACTTGTTTCAGGCAACA
MYH11	GTCCAGGAGATGAGGCAGAAAC	GTCTGCGTTCTCTTTCTCCAGC
COL1A1	GATTCCCTGGACCTAAAGGTGC	AGCCTCTCCATCTTTGCCAGCA
ACTA	CTATGCCTCTGGACGCACAACT	CAGATCCAGACGCATGATGGCA
PDGFRA	GACTTTCGCCAAAGTGGAGGAG	AGCCACCGTGAGTTCAGAACGC
PDGFRB	AGGACAAACCGTACCTTGGGTGACT	CAGTTCTGACACGTACCGGGTCTC
CNN1	CTGGCTGCAGCTTATTGATG	CTGAGAGAGTGGATCGAGGG
INTGR	AGAAGCTCAAGCCAGAGG	GCATCTGTGGAAAACACC
RPLPO (housekeeping gene)	AGCCCAGAACACTGGTCTC	ACTCAGGATTTCAATGGTGCC
GAPDH (housekeeping gene)	TGCACCACCAACTGCTTAGC	GGCATGGACTGTGGTCATGAG

Supplemental Figures

A

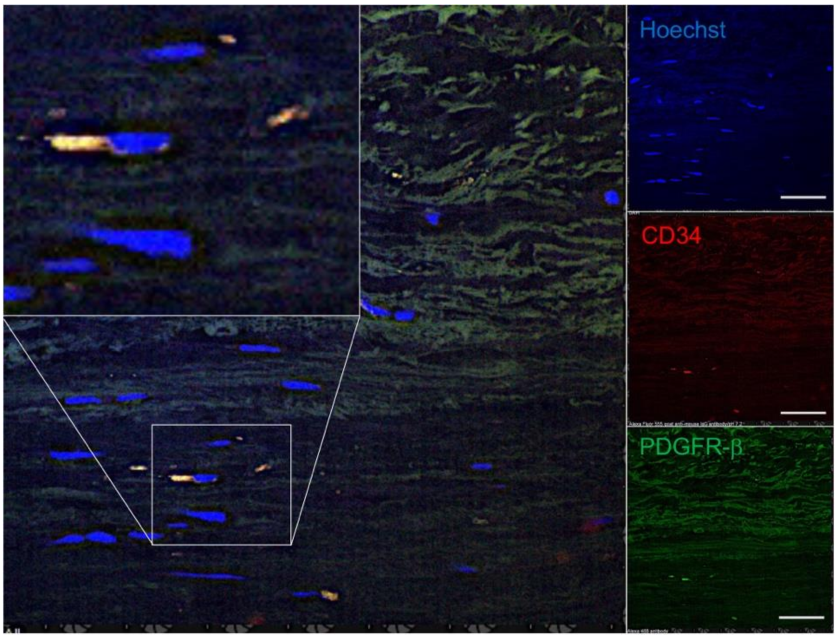

B

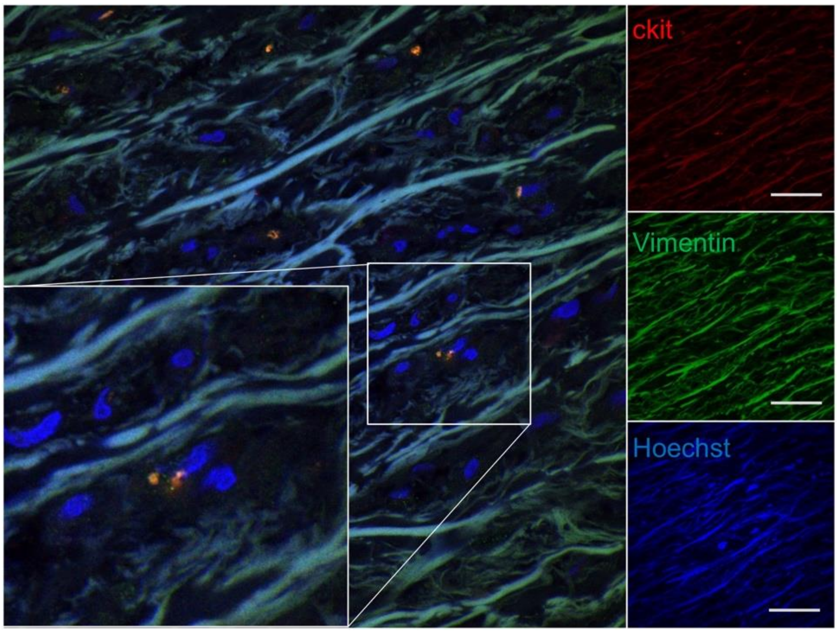

Supplemental Figure S1. CD34, ckit, vimentin and PDGFR-β staining confirmed aortic TC in TAA. Double staining of well-known aortic TC markers in **(A)** CD34 (red) and PDGFR-β (green), and **(B)** ckit (red) and vimentin (green), were done to identified TC specificity. Scalebar, 50 µm.

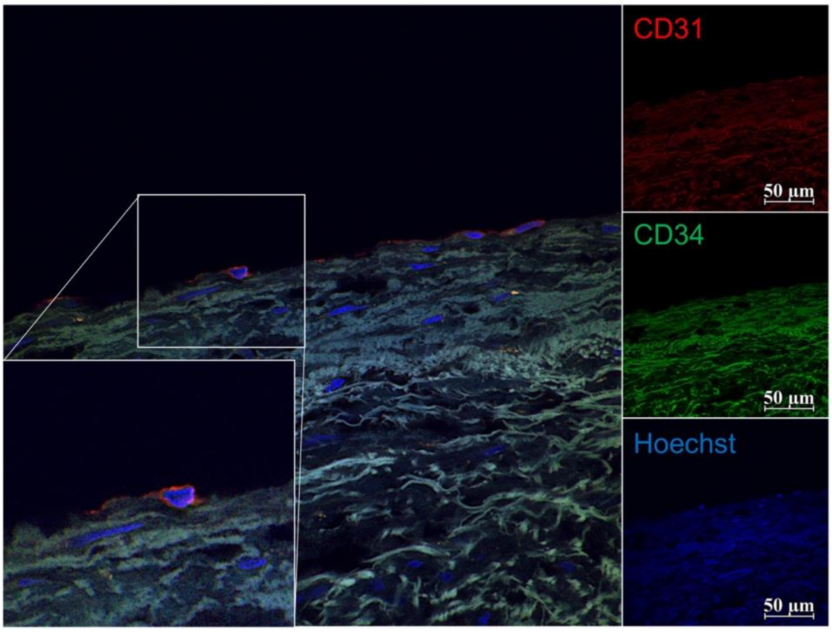

Supplemental Figure S2. Validation of TC specificity by CD34/CD31 double staining. CD34 negative and CD31 positive immunostaining were also described for vasa vasorum or endothelial cell population and should be distinct from aortic TCs. The lack of endothelial marker CD31 were performed to validate aortic TC specificity. Red, CD31; green, CD34; blue, nuclei (Hoechst). Scalebar, 50 µm.

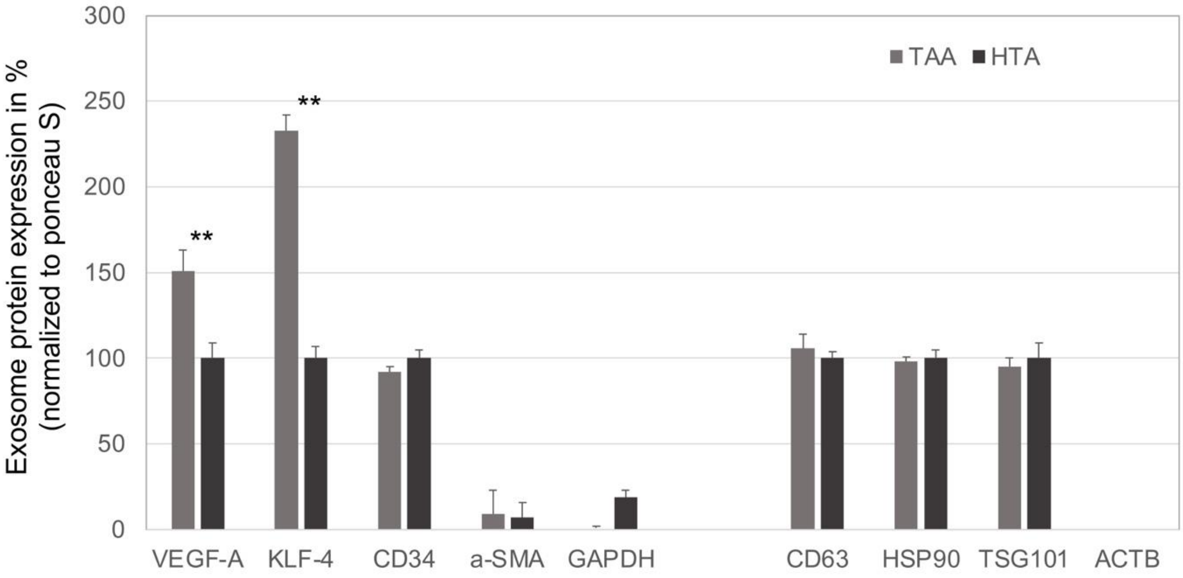

Supplemental Figure S3. Exosome-specific soluble factors VEGF and KLF-4 were increased in exosomes derived from TAA-TCs (gray bars) compared to HTA-TCs (black bars) samples. Soluble factors VEGF-A, KLF-4, CD34 and α-SMA, as well as surface proteins, CD63, HSP90 and TSG101, were analyzed by Western Blot (Figure 2H-I). ACTB and GAPDH were used as loading control. S-Figure 1 shows statistical calculation of protein expression after normalization to ponceau S signals. **, $p < 0.01$. Data are mean ± SD of three independent experiments.
